# Emerging long-term trends and interdecadal cycles in Antarctic polynyas

**DOI:** 10.1073/pnas.2321595121

**Published:** 2024-03-04

**Authors:** Grant A. Duffy, Fabien Montiel, Ariaan Purich, Ceridwen I. Fraser

**Affiliations:** ^a^Department of Marine Science, University of Otago, Dunedin 9054, New Zealand; ^b^Department of Mathematics and Statistics, University of Otago, Dunedin 9054, New Zealand; ^c^School of Earth, Atmosphere and Environment, and Australian Research Council Special Research Initiative for Securing Antarctica’s Environmental Future, Monash University, Clayton, Kulin Nations, VIC 3800, Australia

**Keywords:** sea ice, Antarctica, polynya, Amundsen Sea Low, Southern Ocean

## Abstract

Polynyas, areas of open water embedded within sea ice, are a key component of ocean–atmosphere interactions that act as hotspots of sea-ice production, bottom-water formation, and primary productivity. The specific drivers of polynya dynamics remain, however, elusive and coupled climate models struggle to replicate Antarctic polynya activity. Here, we leverage a 44-y time series of Antarctic sea ice to elucidate long-term trends. We identify Antarctic-wide linear increases and a hitherto undescribed cyclical pattern of polynya activity across the Ross Sea region that potentially arises from interactions between the Amundsen Sea Low and Southern Annular Mode. While their specific drivers remain unknown, identifying these emerging patterns augments our capacity to understand the processes that influence sea ice. As we enter a potentially new age of Antarctic sea ice, this advance in understanding will, in turn, lead to more accurate predictions of environmental change, and its implications for Antarctic ecosystems.

Polynyas provide essential pockets of ice-free habitat for Antarctic nearshore ecosystems. However, in concert with a potentially novel sea-ice regime (1), and increasing coastal exposure (2) and human activity (3) around the Antarctic Peninsula, larger or longer-lived polynyas could leave Antarctic coasts more prone to ocean-driven erosion and incursions by non-native species whose establishment is currently impeded by sea ice. Polynyas are poorly reproduced by current-generation-coupled climate models (4), and accurate simulations of Antarctic sea ice are lacking (5). This inability to replicate polynya activity reflects shortcomings around how time-lagged relationships are incorporated into models (6) and may be part of the more widespread discrepancies between modeled and observed trends in Antarctic sea ice (5). Understanding of polynya dynamics can, therefore, provide insight into the processes that drive polynya formation while also addressing limitations in sea-ice and climate modeling more broadly.

The 44-y time series derived from passive microwave remote sensing (7) provides a unique opportunity to examine long-term patterns of sea-ice concentration that result from processes acting at interdecadal scales (8). Using these data, we identified trends in, and examined the potential drivers of, polynya area variability across Antarctica.

## Results and Discussion

**Fig. 1.**
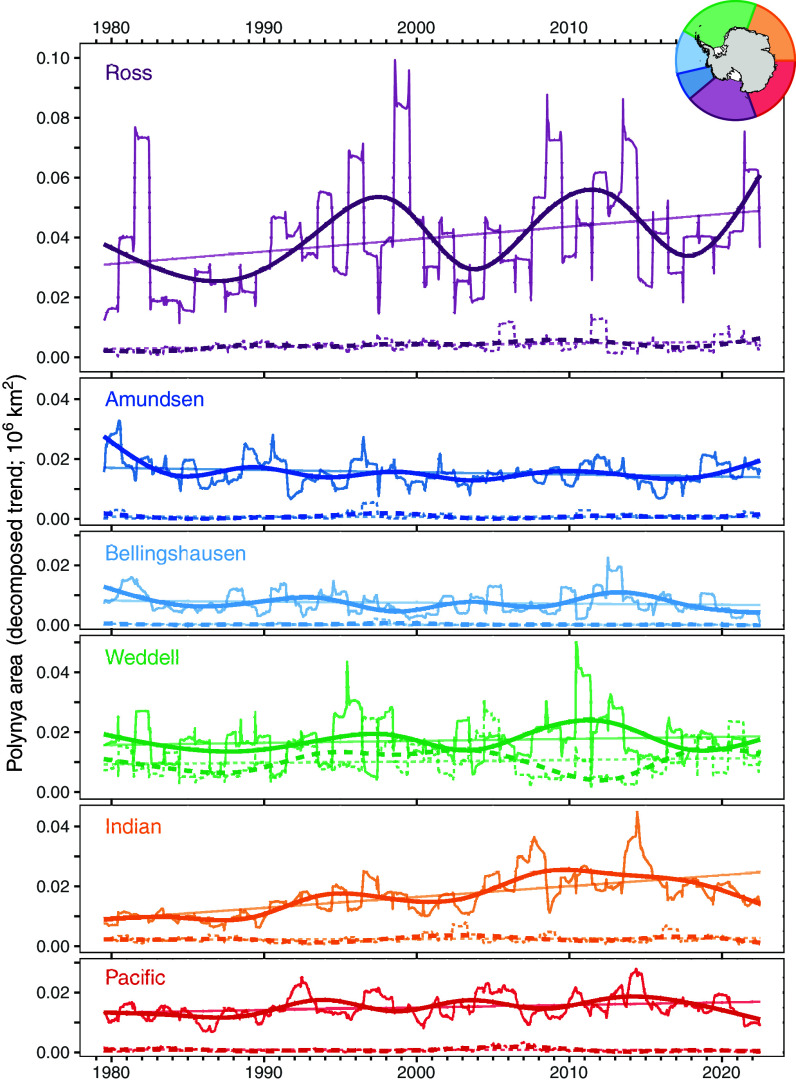
Decomposed trend in polynya area from 1979 to 2022 for each Antarctic sector (see *Inset* map). Straight lines indicate statistically significant (*P* < 0.05) linear trend, smoothed lines indicate the fit of a generalized additive model using time (via a cubic spline smoother) as the sole predictor. Solid and dashed lines indicate coastal and oceanic polynyas, respectively.

**Fig. 2.**
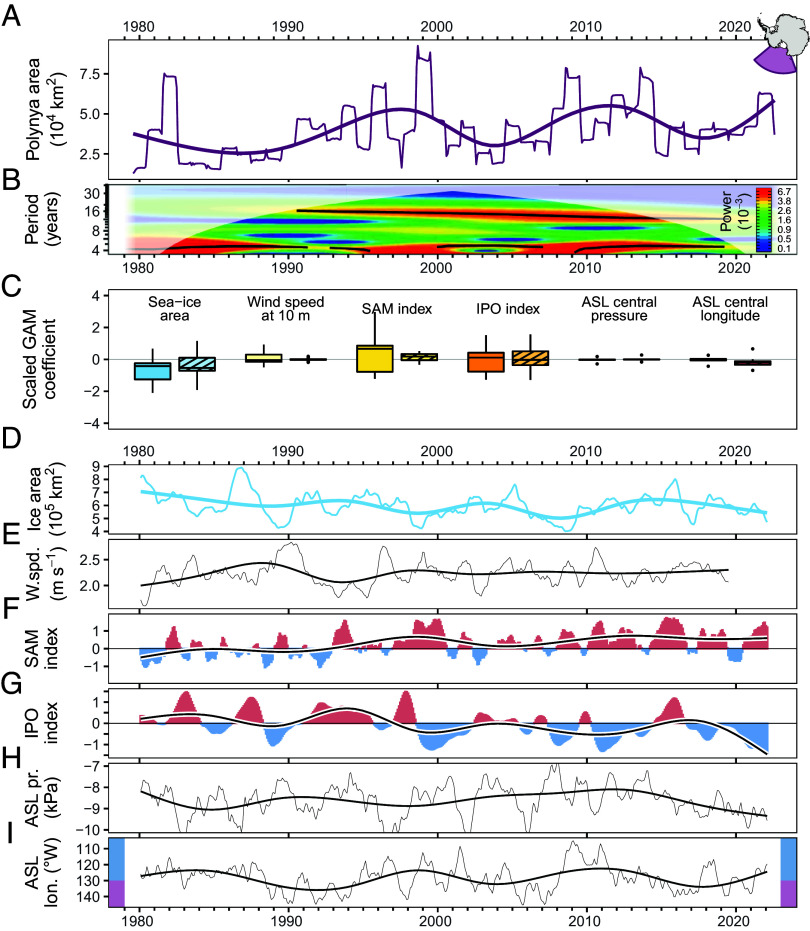
Trend in coastal polynya area in the Ross sector (*A*) and associated wavelet power spectrum (*B*), scaled coefficient (an indicator of relative effect size) distributions for each statistically significant (*P* < 0.05) predictor in non-lagged (solid fill) and 3-mo lagged (hatched fill) GAM [see *SI Appendix*, *Extended Methods*; (*C*)], and decomposed trends for each variable (*D*–*I*). Smoothed thick lines show fits of a GAM using time (via a cubic spline smoother) as the sole predictor. The wavelet power spectrum (*B*) was calculated from coastal polynya area time series data prior to decomposition; black lines indicate wavelet ridges and white shading indicates areas which may be contaminated by edge effects. Colored bars on (*I*) indicate longitudinal limits of the Ross (purple) and Amundsen (blue) sectors.

Over the past 44 y, there has been a positive linear increase in area of Antarctic polynyas across all but the Amundsen and Bellingshausen sectors (Fig. 1). This increase is correlated with an increasingly positive Southern Annular Mode (SAM) index and more negative Interdecadal Pacific Oscillation (IPO) index in recent decades (Fig. 2). Expansion is most pronounced in coastal polynyas (Fig. 1, solid lines) and occurs despite little increase in the number of polynyas (i.e., coastal polynyas are increasing in size). Meanwhile, small but significant increases in area have occurred for oceanic polynyas [Fig. 1, dashed lines; noting that the annual reoccurrence of the c. 250,000 km^2^ Weddell Sea Polynya from 1974 to 1976 (9) is not captured in the 1979 to 2022 time series analyzed here]. Sea-ice concentration in the Amundsen and Bellingshausen sectors has undergone a long-term decline over the satellite period (8) and the west coast of the Antarctic Peninsula has experienced an average increase in coastal exposure of more than 2 d per year over the last 50 y (2). Thus, declines in polynya area in these regions (Fig. 1) reflect increases in ice-free patches connected to the Southern Ocean rather than increases in uninterrupted sea ice. The Antarctic Peninsula has been identified previously as a potential hotspot for the future establishment of terrestrial non-native species (3). Reductions in sea-ice coverage could extend the threat of invasion to coastal marine systems by providing more pathways for rafts of non-native species to reach Antarctic coasts, and by creating ice-free, wave-exposed habitat that is favored by many shallow-water sub-Antarctic species (10).

Polynyas are inherently linked to the sea-ice area, but polynya area does not precisely follow historic growth and recent severe contraction (1, 5, 8) of Antarctic sea ice (Fig. 2). This partial decoupling is further evidenced by the presence of a prominent ~16-y cycle in coastal polynyas in the Ross sector (Fig. 2*A*). Variance in trends of coastal polynya area across the Ross sector can be partially explained (adjusted R^2^ = 0.477) by a range of factors (Fig. 2*C*) suggesting that this pattern is an emergent property of the direct (sea-ice area, wind speed at 10 m; Fig. 2 *D* and *E*) and indirect (SAM, IPO, Amundsen Sea Low indices) predictors examined in our analyses. The periodicity observed in the polynya area across the Ross sector best aligns with prolonged periods skewed toward a positive SAM (Fig. 2*F*) and a more eastward Amundsen Sea Low (ASL; Fig. 2*I*). Wavelet power spectra of the SAM and the ASL longitude time series were statistically equivalent to the Ross sector polynya area spectrum shown in Fig. 2*B* (±6-mo and ±18-mo equivalence margins, respectively; *SI Appendix*, *Extended Methods*), while spectra for all other predictors were not equivalent at margins <36 mo. A specific relationship between the SAM and coastal polynyas has not been previously established but positive SAM anomalies have been observed to precede the opening of oceanic polynyas (e.g., ref. 11). Decadal changes in the mean state of the SAM, in combination with high-latitude effects of El Niño–Southern Oscillation (captured in our analyses via the IPO which closely mirrors the decomposed Southern Oscillation Index), are also associated with shifts in the timing of sea-ice retreat and advance (12).

Anomalies in autumn sea-ice extent across the Ross Sea, which contains the largest regular coastal polynya in Antarctica (the Ross Ice Shelf Polynya, one of several coastal polynyas in the Ross sector) and accounts for ~60% of Antarctic-wide autumnal sea-ice variability, have been linked to zonal wind anomalies of the preceding spring (6). More recently, the size and position of the Ross Ice Shelf Polynya was found to exhibit a time-lagged relationship with sea level pressure and the magnitude and position of the ASL; a deeper and more eastward ASL in late spring often precedes a larger and more eastward polynya the following summer (13). This pattern, resulting from the offshore transport of sea ice by strengthened southerly winds and subsequent changes to surface heat flux, has since been described in other coastal polynyas across the Ross sector (14). In our analyses, years of peak polynya area coincide with periods where the central position of the ASL is generally located further east (Fig. 2*I*). Incorporating a time lag of 3 mo between polynya area and all predictor variables explains a similar amount of variance (adjusted R^2^ = 0.449) while indicating a larger relative effect of ASL longitude than is observed in the non-lagged model (Fig. 2*C*). Ultimately, however, the drivers of the oscillatory pattern observed here, and whether or not this pattern persists under contemporary sea-ice regimes (1), are difficult to identify confidently as the full sea-ice dataset captures only two clearly delineated cycles (Figs. 1 and 2).

Examining the longer, 68-y record from meteorological stations, a 16-y periodicity has been observed in winter air temperatures on the Antarctic Peninsula (15); peaks in Ross sector polynya area described here broadly coincide with transitional years where station conditions were switching from negative to positive temperature anomalies. This was closely correlated with the sea-level pressure anomaly off the eastern coast of South America, but not sea surface temperatures, indicating that atmospheric rather than oceanic processes were driving these cycles (15). The same pattern was not found at stations located further south (15), but our results suggest that the processes driving it may be more widespread, or at least linked to, processes influencing sea ice across West Antarctica and as far afield as the Ross Sea. The strong influence of the ASL on wind speed anomalies extends across West Antarctica (13, 14), with the ASL’s central longitude moving between the Ross and Amundsen sectors (Fig. 2*I*). Therefore, if the same processes are at work, and our hypothesis that the ASL is a key component of the Ross sector polynya cycle described here is correct, a comparable signal in Antarctic Peninsula meteorological records aligns with expectations.

Our inferences around the drivers of the oscillation in coastal polynya area in the Ross sector (Fig. 2), a region comprising a fifth of the Antarctic region’s geographic area but accounting for more than half of overall sea-ice variability (6), provide a fundamental step toward a better understanding of polynya dynamics and the climatic and oceanic processes that shape sea ice. Initially, this could improve regional models and projections of sea-ice decline (5, 8) and coastal exposure (2), with the longer-term potential to better incorporate the influence and response of Antarctic sea ice into global-scale models (4). Outputs from these models are essential when forecasting the threat posed by non-native species (3) and, with sea ice acting as a de facto barrier against coastal biological invasions, accurate predictions of sea ice and polynya area and extent are needed to understand how Antarctic nearshore ecosystems will be impacted by ongoing environmental change.

## Materials and Methods

Daily sea-ice concentration data (1979 to 2022 inclusive; 8) were binarized using a ≤50% threshold. A flood filling algorithm was applied; open water patches not connected to ocean were polygonised to create discrete polynya geometries and assigned to an Antarctic sector; any that adjoined the coastline were identified as “coastal.”

Total polynya area was calculated for each day. Wavelet power spectra were computed via a Morlet function. A moving average (quarterly symmetric window) was used to decompose the timeseries into trend, multiplicative seasonal, and random components. Generalized additive models (GAM) were used to examine correlations between direct (sea-ice area, wind) and indirect (climate indices) factors on patterns of polynya area.

## Supplementary Material

Appendix 01 (PDF)

## Data Availability

Geospatial databases data have been deposited in Figshare (10.6084/m9.figshare.24768654). Previously published data were used for this work (7).
