# Supplementary material for: Emerging long-term trends and interdecadal cycles in Antarctic polynyas
Source: Proc Natl Acad Sci U S A. 2024 Mar 4;121(11):e2321595121. doi: 10.1073/pnas.2321595121 (PMC10945784; doi:10.1073/pnas.2321595121)
Supplement: Supplementary file 1 — Appendix 01 (PDF) [file pnas.2321595121.sapp.pdf]

PNAS

Supporting Information for

Emerging long-term trends and interdecadal cycles in Antarctic polynyas

Grant A. Duffy[a]*, Fabien Montiel[b], Ariaan Purich[c], Ceridwen I. Fraser[a]
[a] Department of Marine Science, University of Otago, Dunedin, New Zealand
[b] Department of Mathematics and Statistics, University of Otago, Dunedin, New Zealand
[c] School of Earth, Atmosphere and Environment, and ARC Special Research Initiative for Securing Antarctica's Environmental Future, Monash University, Clayton, Kulin Nations, Australia.

* Corresponding author
Email: grant.duffy@otago.ac.nz

This PDF file includes:

Extended Methods

Extended Methods

Satellite remote sensing is well-suited for monitoring sea-ice extent and area and has been applied previously to identify polynyas [e.g. (1–3)]. The challenges of accessing polynyas, coupled with their sometimes-expansive scale, means that remote sensing is often the only option for polynya surveillance. This is particularly true for oceanic polynyas whose occurrence is more sporadic than their coastal counterparts (4, 5). Passive microwave radiometers can provide broad-scale assessments of sea ice, albeit at coarse spatial resolutions (6). Meanwhile, high-resolution data products from optical sensors can allow us to finely map and monitor smaller scale features [e.g. 7, 8] but this comes with trade-offs including shorter time series and a dependence on cloud-free conditions. Analyses of high-resolution data at oceanwide scales can also be computationally demanding and while identifying coastal polynyas [e.g. (9)] involves surveying a relatively narrow search window (i.e. only the sea ice in proximity to the coast), expanding these analyses to also search for oceanic polynyas would be more demanding. Cloud-penetrating coarser resolution products therefore provide a viable alternative that can be used as the first step in identifying transient polynyas for more detailed in-depth study. Furthermore, the 40+ year continuous time series available for sea-ice monitoring via passive microwave sensors can give us macro-scale insight into sea-ice patterns and trends, which could enable the identification of interactions amongst modes, dipoles, and oscillations at interdecadal scales (10).

All full-year (1979-2022 inclusive; total 44 years) sea-ice concentration data were retrieved from the European Organisation for the Exploitation of Meteorological Satellites (EUMETSAT) Ocean and Sea Ice Satellite Application Facility Global Sea Ice Concentration Climate Data Record [CDR; v2.0; (11, 12)], which combines observations collected by multiple satellites equipped with either Scanning Multi-channel Microwave Radiometer (SMMR), Special Sensor Microwave/Imager (SSM/I), or Special Sensor Microwave Imager/Sounder (SSMIS) instruments with a post-processing spatial resolution of 625 km^2 (25 x 25 km). Differing approaches to standardisation, intercalibration amongst instruments, and sea-ice concentration calculations may contribute to biases in the time series and small discrepancies when compared against other sea-ice concentration CDRs (13) (e.g. those produced by the NASA Goddard Spaceflight Center) but all data and algorithms have, nevertheless, passed validation and quality assurance (12). Data from more recently deployed microwave radiometers can produce data at higher spatial resolutions (2) but with the trade-off of substantially shortened time series. Thin surface ice along coastlines and on the periphery of polynyas may also lead to underestimates of polynya area when calculated using sea-ice concentration data (13). Using ice thickness data in place of, or in addition to, concentration measurements has been proposed to address this (3) but the length of the ice concentration time series is unmatched (6, 10) and it is the most compatible variable for comparisons against climate model outputs (14).

Sea-ice concentration data were binarized using a thresholding method at ≤ 50 % sea-ice concentration. A range of sea-ice concentration thresholds have been used previously to identify open water [e.g. (2, 9, 14, 15)]. We opted to use a 50 % threshold to strike a balance between capturing relatively small ice-free patches (i.e. cells with higher sea-ice concentration values) and being confident that we are accurately identifying open water (i.e. cells with low sea-ice concentration values). In addition, all analyses were replicated using ≤ 30 % and ≤ 70 % thresholds, which did not substantially change the patterns observed and describe here. Datasets using each threshold are available at 10.6084/m9.figshare.24768654.

A flood filling algorithm was run across each daily image, filling all pixels connected to the open Southern Ocean. Any remaining regions of open water were identified as embedded patches of open water (either bounded wholly by sea ice, or by sea ice and the Antarctic coastline). Connected patches were polygonised to create discrete geometries. Each discrete geometry was given a unique identifier for each time point at which it was identified. Forward and backward temporal connectivity amongst these geometries was identified using the spatial overlap of polygons from temporally adjacent daily data. Immediately adjacent cells (including on the diagonal) of the previous or next day were also considered to be overlapping for this purpose.

The generally accepted criteria whereby a patch of open water embedded in sea ice is classified as a polynya is that the opening is extensive (“tens to tens of thousands of square kilometres”), persistent, and recurrent (5). Due to the resolution of data used, all embedded patches of open water identified here were at least 625 km^2 and so meet the first criterion. Ascertaining persistence, especially for smaller ice-free patches, could, however, be complicated by sea-ice movement as our method for linking patches through time only connects patches if they are directly adjacent in space and time. Meanwhile, recurrence is predominantly a feature of coastal polynyas with oceanic polynyas occurring much more sporadically (4, 5). Furthermore, even some named coastal polynyas have occurred only once and persisted for relatively short periods [e.g. Taylor Glacier and Paulding Bay Polynyas, (5), Lützoh-Holm Bay Polynya (16)]. Therefore, for the purposes of our analyses and in all daily calculations of polynya area, ice-free patches of all longevities and sizes were considered to be polynyas rather than choosing an arbitrary threshold that could bias analyses.

Major polynyas were identified based on their largest area across the dataset. Area for every polynya was calculated and polynyas were then sorted by area and date. Starting with the largest and most recent, polynyas were iterated over and all smaller polynyas which intersected (i.e. overlapped) the major polynya (spatially and temporally based on forward and backward connectivity described above) were identified as sub-polynyas and associated with their respective major polynya via a text field attribute (i.e. linked categorically rather than merging geometries so as not to affect subsequent counts). This process was continued along the size-sorted list of polynyas until all polynyas were associated with a major polynya or identified as a major polynya themselves. To discriminate between coastal and open water polynya, a 5 km spatial buffer was applied to the EUMETSAT landmask (i.e. the Antarctic coastline, including ice shelves, at the same 25 x 25 km resolution of the sea-ice data). All major polynyas that intersected this coastal buffer were identified as ‘coastal’. Major polynyas were further classified by Antarctic sector (Weddell, Indian, Pacific, Ross, Amundsen, or Bellingshausen) following a modified version of the sectors defined by Parkinson [(10); Parkinson’s original Amundsen sector was sub-divided into Amundsen and Bellinghasusen sectors]. Any major polynya which spanned two sectors was assigned to the sector containing its largest area. Sub-polynyas inherited these attributes from the major polynya of which they were a part of.

The final polynya inventory was manually compared against existing shorter-term maps and inventories (1, 5, 9, 16–21) to confirm that the major polynyas identified here aligned with locations and timings of previously identified coastal and oceanic polynyas across the Antarctic region. This manual inspection confirmed that all regular coastal polynyas and all previously identified oceanic polynyas (such as the Weddell, Maud Rise, and Cosmonaut Sea Polynyas) in the past 44 years were replicated in our inventory. Differences in the area and longevity of polynyas can be attributed to the varying methods used to identify and delineate polynya boundaries [(16); e.g. data source or ice-concertation threshold used]. Smaller polynyas are rarely the subject of intensive study, so these smaller ice-free patches were more difficult to compare against existing literature.

Total polynya area was calculated for each day in the time series. A Morlet wavelet function (22–24) was used to compute a wavelet power spectrum from raw (i.e. not detrended) polynya area data and identify spectral signatures occurring at frequencies between three and 44 years. A moving average approach was then used to decompose polynya area data into trend, multiplicative seasonal, and random components. A simple linear regression (area ~ date) was used to identify long-term linear trends while a generalised additive model (GAM), fitted using the same formula but with a cubic spline smoother applied to the predictor, was used to identify cyclical trends.

To identify which co-variates best explained monthly variance in polynya area across the Ross sector, additional GAMs were fitted. The first model used the monthly decomposed trend in Ross sector polynya area as the dependent variable and the respective monthly decomposed trends of

Ross sector sea-ice area, Ross sector wind speed at 10 m, the Southern Annular Mode (SAM) index, the Tripole Index for the Interdecadal Pacific Oscillation (IPO), and central pressure and relative longitudinal position of the Amundsen Sea Low (ASL) as predictor variables. The Southern Oscillation Index (SOI), Indian Ocean Dipole (IOD) index, wind direction at 10 m, air temperature at 2 m and sea level pressure were also considered as predictors but were removed from the model due to concurvity with the remaining predictors. In particular, decomposed trends of the IPO and SOI were closely anti-correlated (i.e. when one was positive the other was equally negative in relative magnitude). All predictor variables were scaled and centred to facilitate comparisons of relative effect sizes and a cubic spline smoother was applied to each predictor for model fitting. In the second model, a three-month offset was used to examine the time-lagged relationship between predictors and polynya area, with an equivalent GAM fitted between this and the abovementioned predictor variables. This three-month offset was identified as the optimal offset for our data based on distributed lag non-linear modelling (25, 26), which examines the relationships between dependent and predictor variables using a range of temporal lags to identify which lag produces the strongest relative explanation of variance. For all variables except wind speed, a lag of 3-4 months was identified as providing the strongest association with polynya area.

To test for equivalence between the frequency of inter-decadal patterns identified in Ross sector polynya area and the frequency of inter-decadal patterns identified in predictor variables used in GAM analyses, we calculated wavelet spectra and applied a two one-sided t-tests (TOST) procedure. First, a Morlet wavelet function (22–24) was used to compute wavelet power spectra for each variable from monthly raw (i.e. not detrended) polynya area and predictor data and identify spectral signatures occurring at frequencies between 10 and 44 years. Next, all temporal frequencies within the uppermost 95 % of their respective wavelet powers (i.e. the most prominent spectral signatures; e.g. the reddest areas indicated on Fig. 2 B) were identified, extracted (one numeric vector for each variable), and used in subsequent equivalence testing. For each predictor variable, the vector of most-powerful frequencies was tested against the vector of frequencies identified for Ross sector polynya area (shown in Fig. 2 B) to determine if the two vectors were statistically equivalent. Equivalence testing was performed using bootstrapped (9999 replicates) TOST procedures (27, 28) with an increasing equivalence margin used to identify the threshold at which vectors could be considered statistically equivalent. Starting with a three-month equivalence margin (i.e. testing if the most-powerful frequencies identified in the spectrum for the polynya timeseries were equivalent to those in the predictor ± three months), equivalence margins were increased at three-month intervals (± three month, ± six months, ± nine months etc.) until equivalence was confirmed by the TOST [i.e. both one-sided t-tests were significant at $p < 0.05$, rejecting the null equivalence hypothesis (27, 28)].

Sea-ice area data for the Ross Sea sector was sourced from the same EUMETSAT CDR used for polynya analyses (11, 12). Wind speed and direction across the Ross sector (between 70 °S and the coast/ice shelf) was calculated from u and v vectors at 10 m retrieved from the ERA5 global reanalysis (29). Air temperature at 2 m and sea-level pressure variables were also retrieved from the ERA5 dataset, but these were not included in the final model. Station-based Southern Annular Mode index data were retrieved from the British Antarctic Survey [(30); https://legacy.bas.ac.uk/met/gjma/sam.html]. Southern Oscillation Index data were retrieved from the NOAA National Centers for Environmental Information (https://www.ncei.noaa.gov/access/monitoring/enso/soi). Unfiltered Tripole Index data for the IPO [(31);; https://psl.noaa.gov/data/timeseries/IPOTPI/] and Indian Ocean Dipole (IOD) Dipole Mode Index data (https://psl.noaa.gov/gcos_wgsp/Timeseries/DMI/) were retrieved from the NOAA Physical Sciences Laboratory. Amundsen Sea Low positional and pressure data were retrieved from v3 of Hosking’s Amundsen Sea Low Index [https://github.com/scotthosking/amundsen-sea-low-index; (32)]

All analyses were performed in R (R Core Team, 2022) using the *dlnm*, *ggplot2*, *imager*, *mgcv*, *patchwork*, *terra*, *TOSTER*, *WaveletComp*, and *xts* packages (33–40, 28).

SI References

1. T. Tamura, K. I. Ohshima, S. Nihashi, Mapping of sea ice production for Antarctic coastal polynyas. *Geophys. Res. Lett.* **35**, L07606 (2008).
2. K. Iwamoto, K. I. Ohshima, T. Tamura, Improved mapping of sea ice production in the Arctic Ocean using AMSR-E thin ice thickness algorithm. *J. Geophys. Res. Oceans* **119**, 3574–3594 (2014).
3. K. I. Ohshima, S. Nihashi, K. Iwamoto, Global view of sea-ice production in polynyas and its linkage to dense/bottom water formation. *Geosci. Lett.* **3**, 13 (2016).
4. M. A. Morales Maqueda, A. J. Willmott, N. R. T. Biggs, Polynya Dynamics: a Review of Observations and Modeling. *Rev. Geophys.* **42** (2004).
5. D. G. Barber, R. A. Massom, "Chapter 1 The Role of Sea Ice in Arctic and Antarctic Polynyas" in *Elsevier Oceanography Series*, (Elsevier, 2007), pp. 1–54.
6. T. Hollands, W. Dierking, Dynamics of the Terra Nova Bay Polynya: The potential of multi-sensor satellite observations. *Rem. Sens. Env.* **187**, 30–48 (2016).
7. S. Paul, S. Willmes, G. Heinemann, Long-term coastal-polynya dynamics in the southern Weddell Sea from MODIS thermal-infrared imagery. *Cryosphere* **9**, 2027–2041 (2015).
8. C. Heuzé, L. Zhou, M. Mohrmann, A. Lemos, Spaceborne infrared imagery for early detection of Weddell Polynya opening. *Cryosphere* **15**, 3401–3421 (2021).
9. S. Nihashi, K. I. Ohshima, Circumpolar mapping of Antarctic coastal polynyas and landfast sea ice: Relationship and variability. *J. Clim.* **28**, 3650–3670 (2015).
10. C. L. Parkinson, A 40-y record reveals gradual Antarctic sea ice increases followed by decreases at rates far exceeding the rates seen in the Arctic. *Proc. Natl. Acad. Sci. U.S.A.* **116**, 14414–14423 (2019).
11. EUMETSAT, EUMETSAT Ocean and Sea Ice Satellite Application Facility, Global sea ice concentration climate data record 1979-2015 (v2.0, 2017), OSI-450 (2022) https:/doi.org/10.15770/EUM_SAF_OSI_0008
12. T. Lavergne, *et al.*, Version 2 of the EUMETSAT OSI SAF and ESA CCI sea-ice concentration climate data records. *Cryosphere* **13**, 49–78 (2019).
13. W. R. Hobbs, *et al.*, A review of recent changes in Southern Ocean sea ice, their drivers and forcings. *Glob. Planet. Change* **143**, 228–250 (2016).
14. M. Mohrmann, C. Heuzé, S. Swart, Southern Ocean polynyas in CMIP6 models. *Cryosphere* **15**, 4281–4313 (2021).
15. S. Morelli, F. Parmiggiani, Wind over Terra Nova Bay (Antarctica) during a polynya event: Eta model simulations and satellite microwave observations. *Eur. Phys. J. Plus* **128**, 135 (2013).
16. K. R. Arrigo, G. L. van Dijken, Phytoplankton dynamics within 37 Antarctic coastal polynya systems. *J. Geophys. Res.* **108**, 3271 (2003).
17. R. A. Massom, P. T. Harris, K. J. Michael, M. J. Potter, The distribution and formative processes of latent-heat polynyas in East Antarctica. *A. Glaciology.* **27**, 420–426 (1998).
18. P. Yager, *et al.*, A carbon budget for the Amundsen Sea Polynya, Antarctica: Estimating net community production and export in a highly productive polar ecosystem. *Elementa* **36** (2016).
19. S. Lee, *et al.*, Evidence of minimal carbon sequestration in the productive Amundsen Sea polynya: Carbon Sequestration in the Amundsen Sea. *Geophys. Res. Lett.* **44**, 7892–7899 (2017).
20. Z. Wei, Z. Zhang, X. Wang, Y. Chen, M. Zhou, The thermodynamic and dynamic control of the sensible heat polynya in the western Cosmonaut Sea. *Deep Sea Res. II* **195**, 105000 (2022).
21. L. Zhou, C. Heuzé, M. Mohrmann, Early winter triggering of the Maud Rise Polynya. *Geophys. Res. Lett.* **49** (2022).
22. J. Morlet, G. Arens, E. Fourgeau, D. Glard, Wave propagation and sampling theory—Part I: Complex signal and scattering in multilayered media. *Geophysics* **47**, 203–221 (1982).
23. J. Morlet, G. Arens, E. Fourgeau, D. Giard, Wave propagation and sampling theory—Part II: Sampling theory and complex waves. *Geophysics* **47**, 222–236 (1982).

24. P. Goupillaud, A. Grossmann, J. Morlet, Cycle-octave and related transforms in seismic signal analysis. *Geoexploration.* **23**, 85–102 (1984).
25. A. Gasparrini, B. Armstrong, M. G. Kenward, Distributed lag non-linear models. *Statist. Med.* **29**, 2224–2234 (2010).
26. A. Gasparrini, Modeling exposure–lag–response associations with distributed lag non-linear models. *Stats. Med.* **33**, 881–899 (2014).
27. D. Lakens, Equivalence Tests: A Practical Primer for *t* Tests, Correlations, and Meta-Analyses. *Soc. Psychol. Pers. Sci.* **8**, 355–362 (2017).
28. A. R. Caldwell, "Exploring Equivalence Testing with the Updated TOSTER R Package" (PsyArXiv, 2022) https:/doi.org/10.31234/osf.io/ty8de
29. H. Hersbach, *et al.*, The ERA5 global reanalysis. *Q. J. R. Meteorol. Soc.* **146** (2020).
30. G. J. Marshall, Trends in the Southern Annular Mode from Observations and Reanalyses. *J. Climate* **16**, 4134–4143 (2003).
31. B. J. Henley, *et al.*, A Tripole Index for the Interdecadal Pacific Oscillation. *Clim. Dyn.* **45**, 3077–3090 (2015).
32. J. S. Hosking, A. Orr, T. J. Bracegirdle, J. Turner, Future circulation changes off West Antarctica: Sensitivity of the Amundsen Sea Low to projected anthropogenic forcing. *Geophys. Res. Lett.* **43**, 367–376 (2016).
33. H. Wickham, *ggplot2: Elegant Graphics for Data Analysis* (Springer, 2009).
34. A. Gasparrini, Distributed Lag Linear and Non-Linear Models in *R* : The Package dlnm. *J. Stat. Soft.* **43** (2011).
35. S. N. Wood, *Generalized Additive Models: An Introduction with R*, 2nd Ed. (Chapman and Hall/CRC, 2017) https:/doi.org/10.1201/9781315370279.
36. A. Roesch, H. Schmidbauer, WaveletComp: Computational Wavelet Analysis (2018).
37. S. Barthelmé, D. Tschumperlé, imager: an R package for image processing based on CImg. *JOSS* **4**, 1012 (2019).
38. T. Lin Pedersen, patchwork: The Composer of Plots (2022).
39. J. A. Ryan, J. M. Ulrich, xts: eXtensible Time Series (2022).
40. R. J. Hijmans, terra: Spatial Data Analysis (2023).
